# Facial Pain as the Initial Presentation of Rhabdomyosarcoma: A Case Report

**DOI:** 10.1002/ccr3.70944

**Published:** 2025-10-17

**Authors:** Najmeh Anbiaee, Atessa Pakfetrat, Shayan Yousefi, Nasrollah Saghravanian, Samaneh Salari

**Affiliations:** ^1^ Department of Oral and Maxillofacial Radiology, Faculty of Dentistry Mashhad University of Medical Sciences (MUMS) Mashhad Iran; ^2^ Department of Oral Medicine Mashhad University of Medical Sciences (MUMS) Mashhad Iran; ^3^ Student Research Committee, Faculty of Dentistry, Mashhad University of Medical Sciences, Iran; ^4^ Department of Pathology, Faculty of Dentistry Mashhad University of Medical Sciences (MUMS) Mashhad Iran

**Keywords:** advanced imaging diagnosis, case report, orofacial pain, persistent idiopathic facial pain (PIFP), rhabdomyosarcoma

## Abstract

Spindle cell/sclerosing rhabdomyosarcoma (RMS) is an uncommon and aggressive neoplasm, especially in adults. This case details a 35‐year‐old male with a 4‐year history of persistent idiopathic facial pain (PIFP), first misdiagnosed owing to the lack of definitive clinical or radiologic evidence. Advanced imaging ultimately identified a malignant tumor in the infratemporal fossa, which was confirmed as rhabdomyosarcoma through histological examination. Owing to the tumor's inaccessibility, surgical removal was unfeasible, and despite chemotherapy, the cancer advanced, resulting in the patient's death. This example highlights the significance of prompt advanced imaging and interdisciplinary cooperation in detecting uncommon cancers that manifest as persistent pain. Given the diagnostic challenge, this case underscores the critical need to consider rhabdomyosarcoma in the differential diagnosis of persistent head and neck pain, especially when conventional evaluations fail to determine a cause. Early recognition and comprehensive diagnostic approaches can improve patient outcomes.

**Summary**

- Facial pain as the initial symptom of rhabdomyosarcoma is rare and can lead to delayed diagnosis.
- Early recognition of persistent, unexplained facial pain as a potential warning sign is crucial for timely intervention and improved patient outcomes.

## 1 Introduction

Rhabdomyosarcoma (RMS) is a malignant tumor of mesenchymal cells that develop into skeletal muscle. It is the most common soft tissue sarcoma in children and presents a treatment challenge due to its aggressiveness and variety [1, 2, 3]. It is the third most prevalent pediatric extracranial solid tumor, with approximately 250 cases diagnosed annually in the United States [4, 5]. It often occurs in the head and neck, genitourinary tract, and extremities. Also, it can occur in the orbit of the eye [4, 6, 7, 8]. The median age of adult patients with Rhabdomyosarcoma varies, with studies reporting median ages ranging from 28 to 59 years [7, 9, 10].

Genetic and histological factors separate Rhabdomyosarcoma into subgroups. The main subtypes include embryonal, alveolar, spindle cell/sclerosing, and pleomorphic rhabdomyosarcoma. Each subtype differs morphologically and molecularly [11, 12]. Biopsies and molecular analysis are needed for accurate diagnosis. MYOD1 and myogenin immunohistochemistry is often used to confirm the rhabdomyoblastic phenotype [11, 13].

Surgery, chemotherapy, and radiation are usually used. Even with advancements, metastatic and recurrent rhabdomyosarcoma patients have a terrible prognosis, especially high‐risk patients [5, 13, 14]. Survival rates are significantly worse for adults compared to children with similar tumors [15].

This report highlights the diagnostic challenge of persistent idiopathic facial pain (PIFP) when standard evaluations fail to identify a cause. A patient with a four‐year history of persistent idiopathic facial pain, worsening after dental procedures, was initially diagnosed with a nonneoplastic condition. However, a malignant tumor in the infratemporal region was later discovered through advanced imaging. The tumor's aggressive nature, including sinus wall erosion, underscores the need for a multidisciplinary approach. This case emphasizes the importance of comprehensive imaging and collaboration between specialties for early diagnosis and improved outcomes.

## 2 Case Presentation

**FIGURE 1:**
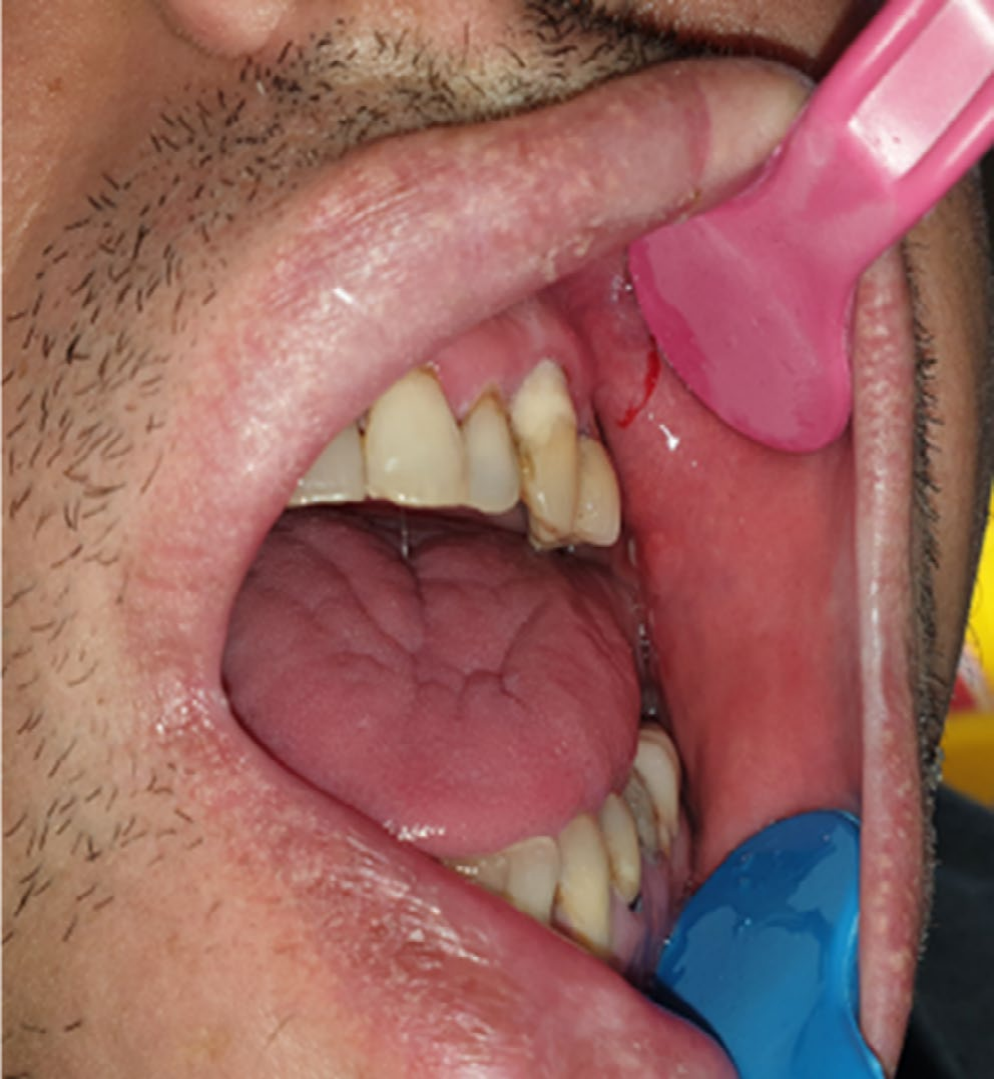
Clinical photograph of the patient.

A 35‐year‐old male patient presented to the Department of Oral Medicine on December 20, 2023, with complaints of severe, persistent pain localized to the left side of his face (Figure 1). The patient reported that the pain had persisted for approximately 4 years, though it significantly worsened 2 months prior when he sought treatment from a general dentist. During this period, the patient underwent root canal therapy on the left maxillary canine and extraction of the first left premolar, followed by curettage of the extraction site. Unfortunately, the interventions did not provide any relief, and the pain progressively intensified postextraction.

The patient was referred to an ear, nose, and throat (ENT) specialist, who performed a full assessment but did not identify any abnormalities. After continued pain, the patient was referred to the Department of Oral Medicine for further evaluation.

Upon presentation, the patient exhibited no limitation in mouth opening, and a thorough intraoral and extraoral examination revealed no abnormal findings. However, the patient complained of significant discomfort upon palpation of the ridge, cheek, and tongue, specifically reporting burning sensations. Additionally, he described episodes of severe pain lasting 2–3 h daily, which radiated to the ear, temple, and eye. These episodes were accompanied by sensitivity to light and sound, dizziness, nausea, vomiting, and a persistent salty taste in the mouth. The patient noted that the pain initially began following a period of significant emotional stress and became exacerbated after the dental extractions. Given the complexity of his symptoms, a provisional diagnosis of persistent idiopathic facial pain (PIFP) was made, and the patient was referred to a neurologist for further management. The patient was prescribed carbamazepine, trifluoperazine, and gabapentin, but no significant improvement was observed after 2 weeks.

## 3 Methods

**FIGURE 2:**
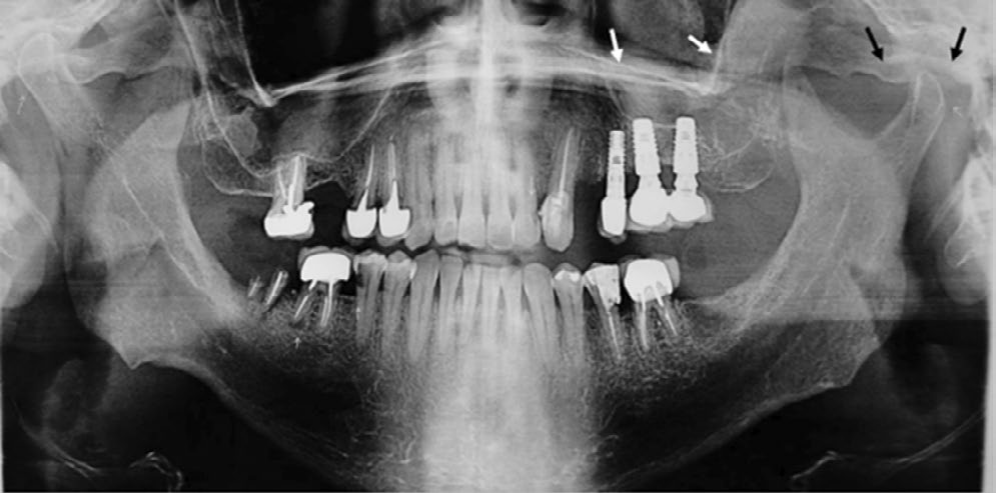
Panoramic radiograph illustrating maxillary sinus opacification and TMJ space widening.

**FIGURE 3:**
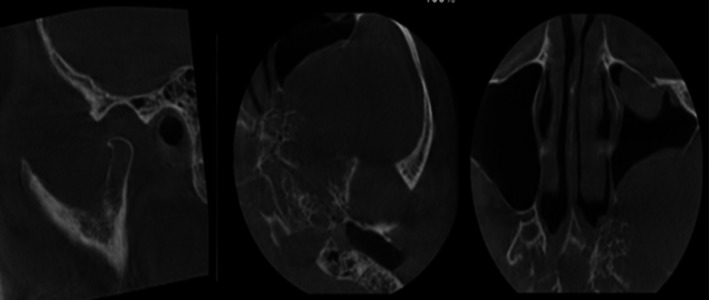
CBCT imaging demonstrating a solid lesion with posterior maxillary sinus wall erosion.

Consequently, the patient was referred to a neurosurgeon for additional evaluation. The OPG images showed fullness of the left maxillary sinus and increased joint space (Figure 2). The CBCT scans revealed an enlarged temporomandibular joint (TMJ) space and the presence of a solid lesion within the temporomandibular space (Figure 3). This lesion had displaced the posterior wall of the left maxillary sinus anteriorly and caused erosion of the sinus wall. Based on these findings, the differential diagnosis included angiofibroma, lymphoma, malignant nerve sheath tumor, squamous cell carcinoma (SCC), and malignant nasopharyngeal carcinoma.

**FIGURE 4:**
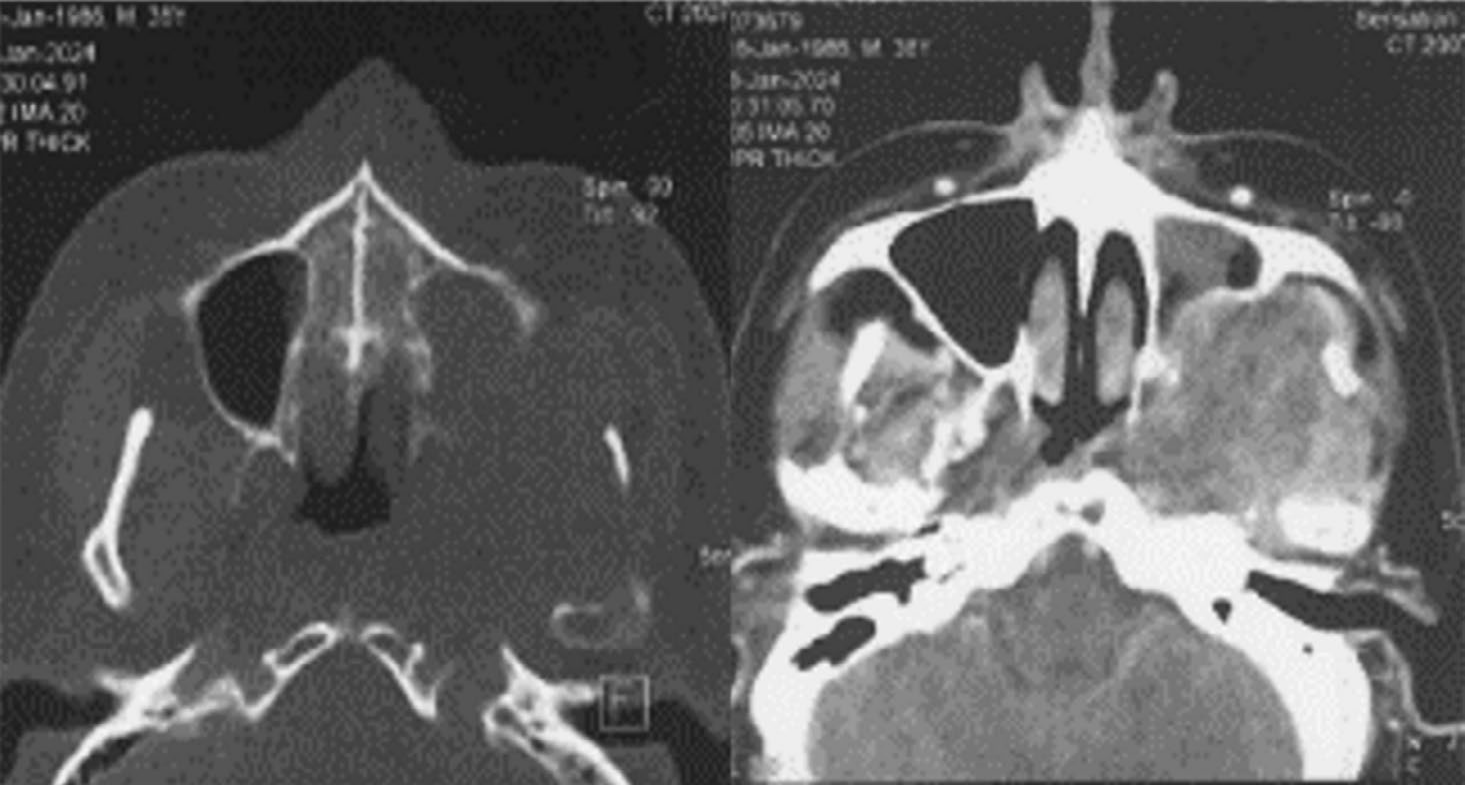
Spiral CT scans revealing the extent of the infratemporal mass and associated bone destruction.

The patient initially underwent a CT scan (Figure 4). Subsequently, an ultrasound‐guided evaluation was considered to examine the salivary glands, thyroid, and neck. However, due to the absence of detectable lesions, performing a biopsy under ultrasound guidance was not feasible. Given this limitation, an MRI was conducted for further assessment. The MRI revealed a heterogeneous solid mass in the left infratemporal fossa, which had eroded and displaced the posterior wall of the maxillary sinus.

**FIGURE 5:**
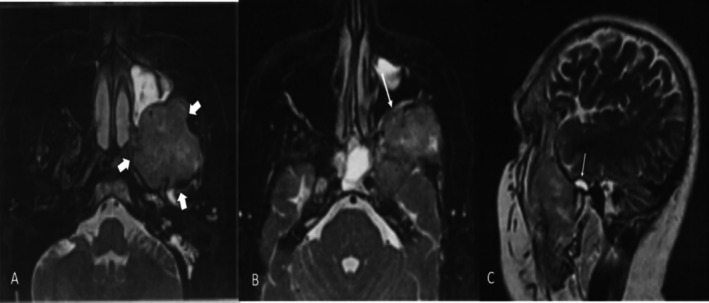
Representative Magnetic Resonance Imaging (MRI) scan of the patient illustrating the radiological appearance of the lesion.

MRI T2 axial and sagittal views showed a medium‐signal, heterogeneous mass occupying the left infratemporal fossa. The lesion caused inflammation, mucositis, and exudate formation within the left maxillary sinus, as well as the temporomandibular joint space. Hyperintense areas within the mass suggested internal hemorrhage or exudate. Moreover, the mass had destructively invaded the posterior wall of the maxillary sinus and penetrated the submucosal layers of the sinus wall (Figure 5).

We reviewed the patient's CBCT, MRI, and CT scans, which confirmed the presence of a mass. The patient was initially referred for biopsy; however, due to accessibility limitations, surgical intervention and biopsy by an oral and maxillofacial surgeon were not feasible. Consequently, the patient was referred for ultrasound‐guided biopsy, but this approach was also deemed impractical. Ultimately, an open surgical biopsy was performed, and the pathological report was as follows.

**FIGURE 6:**
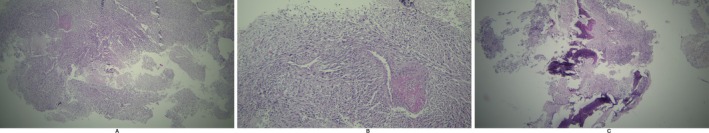
Histopathological section of the tumor stained with Hematoxylin and Eosin (H&E) for routine microscopic examination.

Histopathological examination of the tumor revealed a malignant neoplasm with a spindle cell morphology, characterized by elongated tumor cells with hyperchromatic nuclei and moderate eosinophilic cytoplasm arranged in intersecting fascicles. The presence of pleomorphic cells and areas of increased mitotic activity suggested an aggressive neoplasm. Immunohistochemical analysis was performed to further characterize the lesion (Figure 6).

The tumor exhibited focal weak positivity for smooth muscle actin (SMA), supporting a mesenchymal origin and indicating partial myogenic differentiation. CD31 was negative, effectively ruling out a vascular endothelial neoplasm. The proliferation index, assessed using Ki‐67, was significantly elevated, suggesting a high mitotic rate and rapid tumor growth. The absence of S100 and SOX10 expression excluded neural and melanocytic differentiation, making diagnoses such as malignant peripheral nerve sheath tumor (MPNST) and melanoma unlikely. Strong positivity for myogenin (MYO) confirmed skeletal muscle differentiation, a key diagnostic feature of rhabdomyosarcoma. Additionally, vimentin showed diffuse positivity, further supporting the mesenchymal nature of the tumor.

## 4 Outcome and Follow‐Up

**FIGURE 7:**
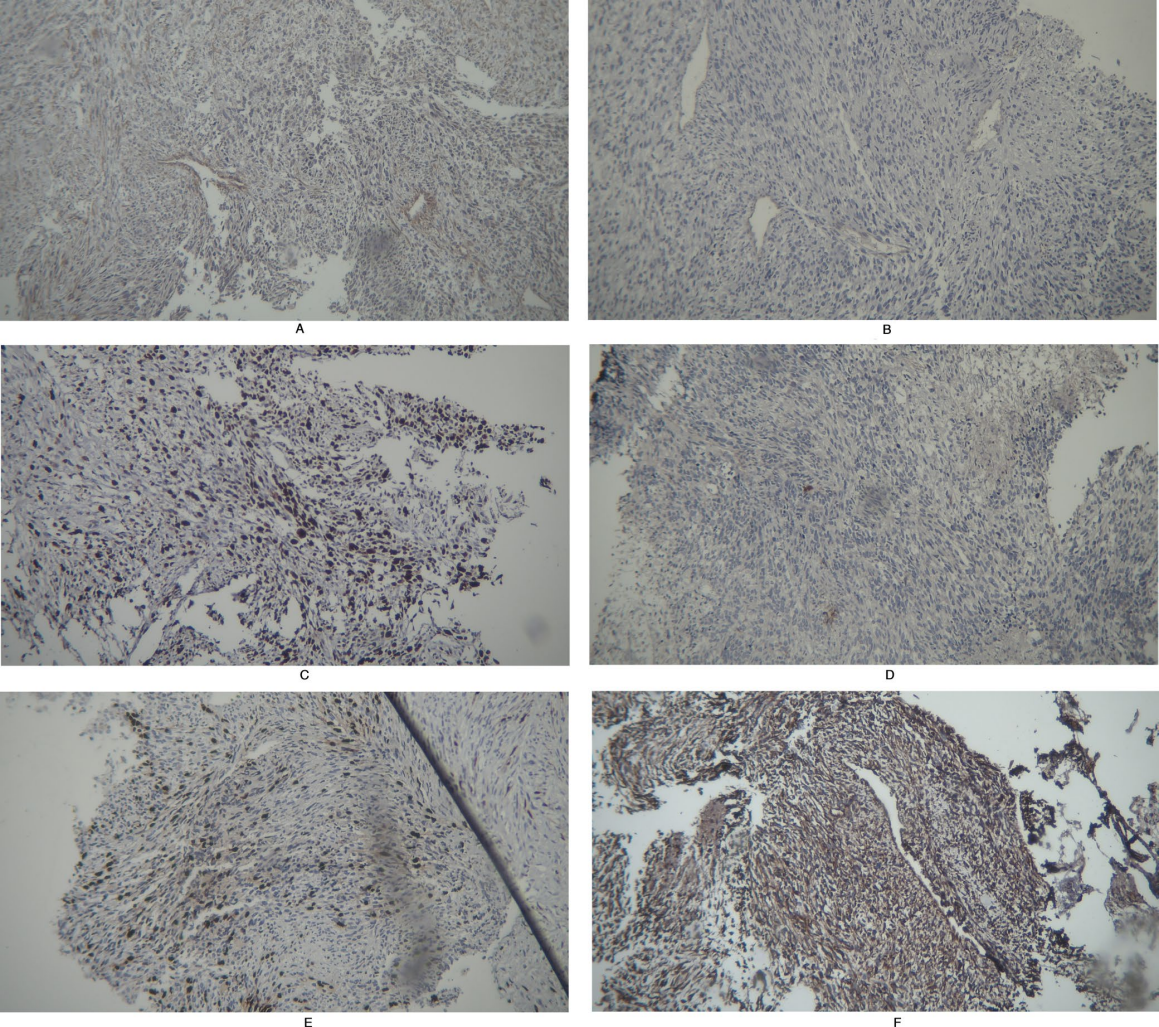
Immunohistochemical staining of the tumor tissue: (A) Smooth Muscle Actin (SMA – Mild positive), (B) CD‐31 (negative), (C) Ki‐67 (positive), (D) S100 (negative), (E) Myogenin (positive), and (F) SOX10 (negative).

Based on the imaging findings, the patient was diagnosed with a malignant tumor, and chemotherapy was initiated, which resulted in a marked reduction in the patient's pain. However, despite the initial improvement in symptoms, the disease proved to be aggressive, and the patient ultimately succumbed to the malignancy. Given the histopathological and immunohistochemical findings, in conjunction with the radiologic and clinical presentation, the diagnosis of spindle cell/sclerosing rhabdomyosarcoma was strongly favored. The tumor's aggressive nature, including its infiltrative growth pattern and high proliferative index, underscores the necessity of a multidisciplinary therapeutic approach involving surgery, chemotherapy, and radiation therapy to improve patient outcomes (Figure 7).

## 5 Discussion

This case presents an unusual and diagnostically challenging instance of spindle cell/sclerosing rhabdomyosarcoma (RMS) in the infratemporal fossa of an adult patient. Rhabdomyosarcoma is a rare cancer arising in skeletal muscle that typically impacts children and young adults [4, 5, 16]. Most cases occurred in pediatric patients (44.4%), predominantly in the para‐meningeal region (57.7%), with the alveolar subtype being the most common (43.2%) [17]. Also, the occurrence of rhabdomyosarcoma in the infratemporal fossa is exceedingly rare, as this deep anatomical area contains intricate neurovascular pathways, complicating diagnosis and treatment. Schwannomas and meningiomas were the most common tumors identified in the infratemporal fossa. The most frequent presenting symptoms included facial hypoesthesia and auricular or preauricular pain [18]. A case series showed that head and neck rhabdomyosarcoma affects the neck, maxillary sinus, nasal cavity, cheek, and infratemporal fossa. Lymph node involvement was common, and distant metastases were observed in some cases, particularly in alveolar and pleomorphic subtypes. Prognosis was generally poor in metastatic cases, while spindle cell/sclerotic rhabdomyosarcoma showed better survival [19].

A major challenge in this case was the atypical presentation of symptoms. The patient presented with facial pain, which initially led to an incorrect diagnosis of persistent idiopathic facial pain (PIFP). The chronic pain, exacerbated after dental extractions, delayed the identification of the underlying malignancy. Persistent Idiopathic Facial Pain (PIFP) is a chronic, daily facial pain without a clear neurological cause. Unlike trigeminal neuralgia, it is persistent, unilateral, and lacks autonomic symptoms. Its cause is unclear, requiring multidisciplinary diagnosis and management [20, 21, 22]. Facial pain is often attributed to neuropathic or musculoskeletal disorders, and malignancies in the infratemporal fossa can remain undetected due to the region's deep location and the lack of overt clinical signs.

The diagnostic intricacy was exacerbated by unremarkable results in initial imaging and discussions with many specialists, including dentists, otolaryngologists, and neurologists. Notwithstanding the advancement of symptoms, the exact nature of the disease remained ambiguous until sophisticated imaging (CBCT, MRI, and CT) was conducted, uncovering a solid tumor with aggressive characteristics, including erosion of the sinus wall.

This example highlights the necessity of evaluating malignancy in cases of persistent, unexplained face pain, particularly when conventional therapies are ineffective. Due to the tumor's aggressive and infiltrative characteristics, a multidisciplinary strategy was needed for diagnosis and treatment.

This case underscores the importance of early advanced imaging and multidisciplinary collaboration in diagnosing rare malignancies like infratemporal fossa rhabdomyosarcoma. Persistent, unexplained facial pain warrants thorough investigation, as delayed diagnosis can lead to poor outcomes. Given the tumor's aggressive nature and complex location, surgical options were limited, making chemotherapy and radiotherapy the primary treatments. The poor prognosis of adult‐onset rhabdomyosarcoma highlights the need for heightened clinical suspicion, prompt imaging, and coordinated specialist care to improve early detection and management.

This case underscores the diagnostic difficulties associated with spindle cell/sclerosing rhabdomyosarcoma (RMS) in an adult patient exhibiting persistent idiopathic facial pain (PIFP). The atypical symptoms and postponed diagnosis underscore the necessity of thorough imaging and interdisciplinary cooperation in intricate cases. The tumor's aggressive characteristics and profound anatomical positioning hindered diagnosis and restricted therapy alternatives. This example highlights the imperative for prompt advanced imaging and increased clinical vigilance in individuals experiencing unexplained face pain. Future innovations in targeted therapy and precision medicine may enhance survival rates for adult‐onset rhabdomyosarcoma.

## Author Contributions

**Najmeh Anbiaee:** conceptualization, investigation, visualization. **Atessa Pakfetrat:** supervision, writing – original draft, writing – review and editing. **Shayan Yousefi:** writing – original draft. **Nasrollah Saghravanian:** conceptualization, investigation, supervision, visualization. **Samaneh Salari:** conceptualization, investigation, visualization.

## Consent

Written informed consent was obtained from the patient for the publication of this case report, including all related clinical details and images.

## Conflicts of Interest

The authors declare no conflicts of interest.

## Data Availability

All data analyzed in this case report is included in the published article. No additional datasets are available.
